# Genome-wide scan for commons SNPs affecting bovine leukemia virus infection level in dairy cattle

**DOI:** 10.1186/s12864-018-4523-2

**Published:** 2018-02-13

**Authors:** Hugo A. Carignano, Dana L. Roldan, María J. Beribe, María A. Raschia, Ariel Amadio, Juan P. Nani, Gerónimo Gutierrez, Irene Alvarez, Karina Trono, Mario A. Poli, Marcos M. Miretti

**Affiliations:** 1 Instituto Nacional de Tecnología Agropecuaria (INTA). Centro de Investigaciones en Ciencias Veterinarias y Agronómicas (CICVyA). Instituto de Genética, B1686 Hurlingham, Argentina; 2 Instituto Nacional de Tecnología Agropecuaria (INTA). Estación Experimental Agropecuaria Pergamino, B2700 Pergamino, Argentina; 3 Instituto Nacional de Tecnología Agropecuaria (INTA). Estación Experimental Agropecuaria Rafaela, S2300, Rafaela, Argentina; 4 Consejo Nacional de Investigaciones Científicas y Técnicas (CONICET), C1033AAJ Ciudad Autónoma de Buenos Aires, Argentina; 5 Instituto Nacional de Tecnología Agropecuaria (INTA). Centro de Investigaciones en Ciencias Veterinarias y Agronómicas (CICVyA). Instituto de Virología, B686 Hurlingham, Argentina; 6 Grupo de Investigación en Genética Aplicada, Instituto de Biología Subtropical (GIGA - IBS), Universidad Nacional de Misiones, N3300 Posadas, Argentina

**Keywords:** Bovine leukemia virus, Level of infection, Whole genome association study

## Abstract

**Background:**

Bovine leukemia virus (BLV) infection is omnipresent in dairy herds causing direct economic losses due to trade restrictions and lymphosarcoma-related deaths. Milk production drops and increase in the culling rate are also relevant and usually neglected. The BLV provirus persists throughout a lifetime and an inter-individual variation is observed in the level of infection (LI) in vivo. High LI is strongly correlated with disease progression and BLV transmission among herd mates. In a context of high prevalence, classical control strategies are economically prohibitive. Alternatively, host genomics studies aiming to dissect *loci* associated with LI are potentially useful tools for genetic selection programs tending to abrogate the viral spreading. The LI was measured through the proviral load (PVL) set–point and white blood cells (WBC) counts. The goals of this work were to gain insight into the contribution of SNPs (bovine 50KSNP panel) on LI variability and to identify genomics regions underlying this trait.

**Results:**

We quantified anti–p24 response and total leukocytes count in peripheral blood from 1800 cows and used these to select 800 individuals with extreme phenotypes in WBCs and PVL. Two case-control genomic association studies using linear mixed models (LMMs) considering population stratification were performed. The proportion of the variance captured by all QC-passed SNPs represented 0.63 (SE ± 0.14) of the phenotypic variance for PVL and 0.56 (SE ± 0.15) for WBCs. Overall, significant associations (Bonferroni’s corrected -log_10_p > 5.94) were shared for both phenotypes by 24 SNPs within the Bovine MHC. Founder haplotypes were used to measure the linkage disequilibrium (LD) extent (r^2^ = 0.22 ± 0.27 at inter-SNP distance of 25−50 kb). The SNPs and LD blocks indicated genes potentially associated with LI in infected cows: i.e. relevant immune response related genes (*DQA1, DRB3, BOLA-A, LTA, LTB, TNF*, *IER3, GRP111, CRISP1*), several genes involved in cell cytoskeletal reorganization (*CD2AP, PKHD1, FLOT1, TUBB5*) and modelling of the extracellular matrix (*TRAM2, TNXB*). Host transcription factors (TFs) were also highlighted (*TFAP2D; ABT1, GCM1, PRRC2A*).

**Conclusions:**

Data obtained represent a step forward to understand the biology of BLV–bovine interaction, and provide genetic information potentially applicable to selective breeding programs.

**Electronic supplementary material:**

The online version of this article (10.1186/s12864-018-4523-2) contains supplementary material, which is available to authorized users.

## Background

The Bovine leukemia virus (BLV) is an oncogenic δ-retrovirus that persistently infects cattle [1]. BLV infection elapses without noticeable symptoms in most of the animals while 30–40% of them evolve into a sub-clinical persistent lymphocytosis (PL) characterized by an expansion of proviral infected B-lymphocytes in peripheral blood increasing white blood cells (WBC) counts (leukocytosis). Less than 10% of infected animals will develop the severe form of the disease leading to lethal lymphomas or lymphosarcoma in various organs [2]. The BLV is transmitted among cows through direct exposure to blood, body fluids or consumption of contaminated colostrum/milk with proviral DNA [3, 4]. In the absence of effective measures controlling BLV transmission, the virus is massively propagated reaching high levels of endemicity. BLV is world-wide distributed. In North America BLV’s prevalence reached 70–85% at farm level [5–7], and in Argentina more than 80% of the lactating cows in the main dairy production area are infected [8]. International trade restrictions of livestock products from affected herds [9] impact on the regional economy, but the total economic loss is significantly higher if we consider milk production dropping and faster culling of asymptomatic BLV carriers compared to BLV free herds [10, 11]. These conditions are emphasised in lymphocytotics animals and also in those with high anti-BLV humoral response [12–16]. Traditional control strategy implies segregating/eliminating infected cows after BLV testing, but this is not economically affordable when the regional prevalence is high [17]. A suitable long term approach to contain endemic livestock diseases, especially those where the therapeutics or preventives treatments failed, is DNA-based breeding for host resistance to infection [18]. Mastitis incidence is currently being incorporated in genomic evaluations for dairy cattle ([19, 20]). A key component to set up host genetic studies is properly defining and recording a consistent phenotype [21, 22]. The high PVL has been associated with progression of the BLV pathogenesis [23, 24], and with enhanced viral dissemination [25]. Previous evidence pointed to DNA polymorphisms in candidates genes (in particular, alleles from the bovine MHC class II *DRB* gene) as contributors to the inter-individual variation in the PVL and PL [26–34]. However, genetic variation for resistance/susceptibility to diseases in cattle is usually polygenic, i.e. susceptibility to paratuberculosis [35, 36], mastitis [37, 38] bovine respiratory disease complex (BRDC) [39] and recently observed in a genome-wide mapping for genetics determinants of BLV incidence [40, 41].

With the final objective in mind of developing (implementing) genomics selection programs to control BLV dissemination it is essential to understand the biological mechanism implicated. In an attempt to address this questions we utilized antibodies anti-p24 as cost-effective predictors of the level of PVL in blood [8] on over 1800 animals, characterized with WBC counts, in order to recruit a population of Holstein and Holstein x Jersey crossbred dairy cows representing extreme phenotypes of level of infection (LI) in vivo, and therefore, with different individual risk for viral transmission. Afterwards, we integrate these phenotypes in a case-control genome-wide association study (GWAS) using the Illumina BovineSNP50v2 BeadChip to investigate genomic regions associated with BLV infection.

## Methods

### Study population

Blood samples were obtained from 1800 Holstein and Holstein x Jersey cows representing 25 half-sisters families from 16 dairy herds located in the central region of Argentina. Farm owners’ consent was obtained before animal sampling. All animals have full pedigree and were over 3 years old at the time of sampling and between 1^st^ and 9^th^ lactation (78.7% concentrated between 2^nd^ and 4^th^ lactation). The procedures followed for extraction and handling of samples were approved by the Institutional Committee for Care and Use of Experimental Animals of the National Institute of Agricultural Technology (CICUAE-INTA) under protocol number 35/2010, and followed the guidelines described in the institutional Manual. Blood samples were collected in heparinized tubes. Plasma and fresh blood were frozen at − 20 °C until used.

Quality and concentration of genomic DNA extracted from whole blood samples using a commercial kit (Blood Genomic DNA AxyPrep™, Axygen Biosciences, Union City, USA) was assessed using a micro−volume spectrophotometer (NanoDrop™ Technologies, Inc. Wilmington, USA). DNA samples were normalized to a minimum concentration of 20 ng/μl.

### Phenotyping

The anti-p24 ELISA assay was used to discriminate BLV infected animals [42], followed by total WBC counts. In a previous study we showed that the individual anti-BLV humoral response is a cost effective indirect measure of infection in vivo [8]. Therefore, animals were first screened by anti-p24 ELISA and then divided into two groups of contrasting phenotypes on the basis of their antibodies level. The LI in selected animals was then corroborated by determining blood PVL set-point.

Serology: The recombinant viral core-p24 protein was used to detect plasma anti-BLV antibodies for each sample as described in [42] Briefly, a ratio sample to positive (S/P), called % of reactivity (% R) is obtained: S/P = [(OD_Sample_ - OD_WS-_)/(OD_WS + 1/7_ - OD_WS-_)] × 100, where OD = optical density, WS- = negative serum, WS + 1/7 = serum international standard weak positive control (diluted 1:7 in negative serum) used as reference value. The % R of all samples tested was indicated as “low” (LR), for records between a cut-off value (25%) [42] and the weak positive serum value (100%); and as “high” (HR) in those samples with measurements higher than 200%.

PVL relative quantification: PVL determination was performed over LR and HR animals by real-time PCR using TaqMan technology as described in [42, 43]. A fragment of BLV *pol* gene (target) was amplified simultaneously with the reference gene *18S*. Fetal Lamb Kidney cells (FLK) containing 4 copies/cell of BLV proviral DNA, in a final concentration of 1% in BLV-free peripheral blood mononuclear cells (PBMCs) were used as internal control. This proportion of BLV-infected cells corresponds to a low level of natural infection [42], proved to be 5% in aleukemics animals [44]. The relative PVL was defined according to the equation described by Pfaffl et al. [45]:

$$ R=\frac{{\left({E}_{target}\right)}^{\Delta \mathrm{Ct}}{target}^{\left( control- sample\right)}}{{\left({E}_{ref}\right)}^{\Delta \mathrm{Ct}}{ref}^{\left( control- sample\right)}} $$

where E = reaction efficiency; Ct = threshold cycle. The control PVL was indicated as 1 and all samples tested were referred to this. The detection limit was 1 infected cell /2000 uninfected cells. The PVL was qualified as “No Detectable” (ND) if a Ct value could not be obtained (below the detection limit), as “low” if R < 1 (LPVL) and as “high” if R > 1 (HPVL).

WBC counts: Total leukocyte count was performed manually using a haemocytometer. Fresh blood was diluted 1:20 in a 1% Gentian Violet and 2% acetic acid solution. Normal WBC counts ​​in BLV-seronegative adult cattle are in the range of 2100 to 11,500 cells/μl (mean: 6800 ± 2 SD) [46].

### Genotyping with the medium density SNPs chip

A total of 970 cows with PVL determination (74% Holstein and 26% Holstein x Jersey), and 29 bulls (24 Holstein and 5 Jersey) where chosen for genotyping. SNP genotyping was performed by GeneSeek (Neogen Corporation Company, Lincoln, NE, USA) using the BovineSNP50 v2 BeadChip. The panel tests 54,609 SNPs spaced 49.4 kb on average (median spacing of 37 kb) [47]. SNPs were mapped to the bovine assembly UMD 3.1 [48]. The SNPs identification was reassigned to its corresponding “rs” catalogued in dbSNP [49] using the SNPchiMp v.3 tool [50].

Quality control (QC) of the genotypic data was carried out using a set of tools and routines provided by PLINK v1.07 [51]. Individuals with a genotype call rate (CR_IND_) < 90% were excluded from further analysis. Mendelian inheritance discordances were checked and set as missing. SNPs with a call rate (CR_SNPs_) < 90%, a deviation from HWE *p* < 1.10^− 08^ and Minor Allele Frequency (MAF) < 0.01 were removed from the study. After the QC, the data set was composed of 44,174 SNPs and 925 individuals (898 cows and 27 bulls).

### Case-control definitions

The case-control phenotypes to conduct GWAS for LI in vivo considered 396 cases (HPVL) and 373 controls (ND/LPVL). The alternate phenotype was defined by the number of circulating white blood cells (WBCs), which is indicative of the presence of PL, a sub-clinical symptom of BLV infection [52], clustering 283 cases (WBC ≥ 11,500 cells/μl) and 611 control cows (WBC < 11,500 cells/μl).

### Linkage disequilibrium

We performed chromosomal haplotypic phase assignment using the software DualPHASEv1.1 included in the PHASEBOOK 2.3 package [53] using the default settings. To calculate the extent of linkage disequilibrium (LD) only maternal founders chromosomes were used. Phasing algorithms allowed the imputation of not assigned genotypes [54] so that the missing genotypes or those set as missing due to Mendelian inconsistencies were imputed. Measure of LD extent was addressed by calculating the correlation of alleles in two *loci* (r^2^ and D’) for each chromosome with Haploview v4.2 [55]. The calculations were performed on all pairs of syntenic SNPs with an inter-marker distance < 1 Mb. The r^2^ measure was then averaged at intervals of 10 kb and plotted versus the inter-SNPs distance.

### Population stratification

We inspected the genealogy of bulls in the sampled families (http://www.sra.org.ar/rrgg/) evidencing a strong co-ancestry among them (Additional file 1: Figure S1). In order to prevent spurious associations due to population substructure we performed a multi-dimensional scaling (MDS) analysis on identity by state (IBS) pairwise based distances matrix as implemented in PLINKv1.07 [51]: 1 − (*IBS*2 + 0.5*IBS*1)/*N*, where *IBS*2 is the number of SNPs in which two individuals share two *IBS* alleles; *IBS*1 is the number of SNPs in which only one allele is shared *IBS* and *N* is the number of markers used. Finally, the distance matrix was partitioned into a number of uncorrelated variables (or Principal Components, PC). The first two PCs were used to visualize differences in ancestry among individuals (Additional file 2: Figure S2).

### Association analysis

The GWAS were performed using LMMs as implemented in GCTA v1.24 [56]. The general model in the observed scale can be written as:

$$ y= Xb+ Zu+e $$

where *y* is a vector n × 1 that contains binaries values for PVL or WBC; *b* is a vector that contains the fixed effects of the *SNP*_*i*_ to be tested for association (considering an additive genetic effect) and the co-variables Age (*A*_*j*_) (j = 3 to 12); Herd (*H*_*k*_) (k = 1 to 16); Lactation Number (*L*_*l*_) (l = 1 to 9); Percentage of Holstein breed (*PH*_*m*_) (m = 0, 0.125, 0.25, 0.50, 0.75 and 1); Bull (*B*_*n*_) (*n* = 1 to 25) and PC derived from the co-ancestry analysis (PC1 – PC8). The random effects included the additive polygenic effect *u* of the animal; and the vector *e* of residuals effects, *X* and *Z* stand for the incidence matrix that relate the values in *y* to the fixed effects in *b* and the random effects in *u*, respectively. A liability threshold model *l* = *Xb* + *g* + *e* was utilized in order to relate observations on the observed scale to liabilities on the unobserved continuous scale. Liability phenotypes are distributed as ~N(0,1), On the liability scale, the random effects included the additive polygenic effect *g* of the animal, distributed ~N(0, σ${}_g{}^2G$), where *G* represents the genetic relationship matrix (GRM) calculated from the proportion of shared alleles among individuals for all QC-passed SNPs; and the vector *e* of residuals effects, distributed ~N(0, σ${}_e{}^2I$)), where I is the identity matrix. Other terms are the same as in the linear model but on the liability scale [57, 58]. The heritability on the scale observed $\left({h}^2=\frac{\sigma_u^2}{\sigma_u^2+{\sigma}_{\varepsilon}^2}\right)$ was transformed to that on the liability scale [58]: ${h}_l^2=\frac{h_0^2\ K\left(1-K\right)}{z^2}$; where ${h}_l^2$ is the heritability on the underlying scale; ${h}_o^2$ is the heritability on the observed scale; *K* is the disease prevalence and *z*is the height of the density function of the standard normal probability at the threshold value (*K*).

This model was utilized to calculate the genetic correlation between PVL and WBC. According to Lee et al. [59] the genetic correlation was defined as$\mathit{\operatorname{cov}}\left({g}_1,{g}_2\right)\overset{\sim }{=}{z}_1\frac{P_1\left(1-{P}_1\right)}{K_1\left(\ 1-{K}_1\right)}{z}_2\frac{P_2\left(1-{P}_2\right)}{K_2\left(\ 1-{K}_2\right)}$ $\mathit{\operatorname{cov}}\left({g}_1^{\ast },{g}_2^{\ast}\right)$; where, *g*is the genetic value on the observed scale, *g*^∗^ is the genetic value on the underlying scale, *z* is the height of the density function of the standard normal probability at the threshold value (*K*), *P* is the proportion of cases in the sample and *K* is the prevalence for features 1 (PVL) and 2 (WBC), respectively.

The association analysis implemented in GCTA v1.24 uses the restricted maximum likelihood method (REML) to solve the equations of LMMs [56, 57].

The genetic variance for PVL and WBCs traits was calculated on the 29 individual autosomes and on the X bovine sexual chromosome. The genetic variance was decomposed into separate chromosomal segments following:

$$ y= Xb+\sum \limits_{i=1}^r{g}_i+e $$

where, *g*_*i*_ is a random genetic effects vector corresponding to the complete genome or a particular chromosome. The phenotypic variance was partitioned so that $$ \mathit{\operatorname{var}}(y)=\sum \limits_{i=1}^r $$$$ {\sigma}_i^2G $$ + $$ {\sigma}_i^2I $$ where $$ {\sigma}_i^2 $$is the variance of the *i*-th genetic factor with its related GRM.

The family-wise error rate was controlled by Bonferroni’s correction α′ ≈ α/n, where α is the significance level of 5% and n is the number of SNPs tested. The α′value represents the threshold that the *p*-value must reach for each test to be considered significant.

### Gene content of the genomic regions identified in the association studies

Gene annotation for BTA 23 (UMD3.1 bovine assembly, [48]) was retrieved using BioMart tools [60]. Firstly, SNPs were assigned to a particular gene if they were located on exons, introns or untranslated regions (UTRs). Secondly, SNPs lying within a 20 kb window upstream and downstream from gene boundaries, aiming to capture promoter/expression regulatory regions [61]. Finally, remaining SNPs were assigned to the closest gene from 20 to 30 kb, within the range of useful average LD values ​​found (r^2^ > 0.2) [62–65]. Gene to SNPs mapping was performed using BedTools package [66]. Gene functional classification based in Gene Ontology terms (GO) was performed using the PANTHER classification system website (http://www.pantherdb.org/) [67].

## Results

**Fig. 1:**
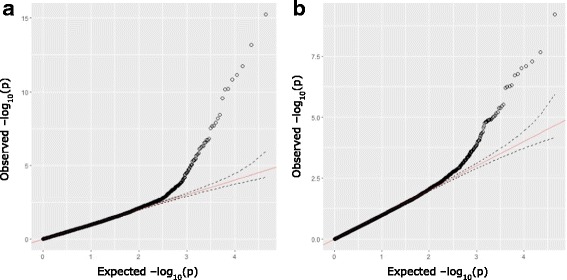
QQ plots. p_obs_ vs p_exp_ obtained from the association mapping using LMMs of 44,174 SNPs for **a**) PVL and **b**) WBCs

Genome-wide distributed SNPs were tested for association with both PVL and WBCs traits using logistic regressions assuming an additive model that incorporated as covariates 8 PCs and other confounding factors Age (A); Herd (H); Lactation number (L); Percentage of Holstein (PH) and Bull (B). The observed statistical values of association were ordered from lowest to highest and plotted against their expected values and genomic inflation factor (λ) was calculated using PLINK. Both for PVL (Additional file 3: Figure S3, λ = 1.21) and WBCs (Additional file 4: Figure S4, λ = 1.30), the data was best fitted with models considering PCs1–8 + A_H_L_PH_B. Given that kinship and/or cryptic relationship might be contributing sources of distortion in the case-control associations we decided to use LMMs as implemented in GCTA v1.24 [56] that incorporates each of QC-passed SNP as a fixed effects and the polygenic animal effect as random. QQ plots showed a proper fit for stratification and a pronounced deviation to the right, which is suggestive of true associations (Fig. 1).

In parallel, genome-wide regression methods incorporating GRM allowed to estimate the fraction of phenotypic variance captured by the common SNPs in the genotyping panel. The heritability (*h*^2^) for PVL was estimated at 0.63 (SE ± 0.14) on the scale of liability, with a prevalence of 40% of cows with high PVL. A *h*^2^_lia_ = 0.56 (SE ± 0.15) to equal prevalence was estimated for a phenotype corresponding to a high WBCs count (> 11,500 cells/μl). Polymorphisms in only three chromosomes explained more than 5% of the phenotypic variation for PVL: *Bos taurus* autosome (BTA) 10 7.1% (SE ± 5.0%), BTA 11 5.8% (SE ± 4.1%) and BTA 23 30.6% (SE ± 7.8%). Similarly, when considering the percentage of variation in WBCs, BTA 11 12.4% (SE ± 6.5%), BTA 21 5.4% (SE ± 5.0%) and BTA 23 24.0% (SE ± 7.2%) autosomes accounted for 74% of the total variance explained by all SNPs (58%).

**Fig. 2:**
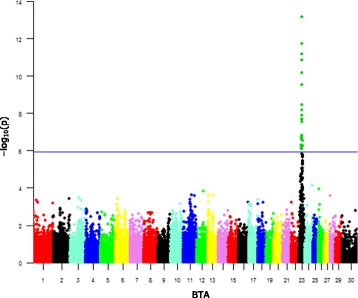
Manhattan plot depicting GWAS results of BLV level of infection using LMMs for PVL. The -log_10_(p) values for each SNP association is represented for each chromosome (BTA) and location within it. The SNPs exceeding the significance threshold according to Bonferroni’s correction (−log_10_(*p*) > 5.94, blue horizontal line) are highlighted in green

**Fig. 3:**
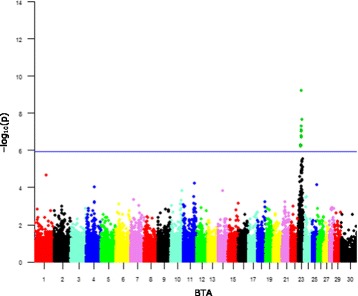
Manhattan plot depicting GWAS results of BLV level of infection using LMMs for WBCs. The -log_10_(p) values for each SNP association is represented by chromosome (BTA) and location within it. The SNPs exceeding the significance threshold according to Bonferroni’s correction (−log_10_(*p*) > 5.94, blue horizontal line) are highlighted in green

The Manhattan plots revealed significantly associated SNPs with HPVL in peripheral blood (Fig. 2) and with a high number of circulating WBCs (Fig. 3).

All SNPs exceeding the significance threshold after Bonferroni’s correction (−log_10_p > 5.94) are located on BTA 23. Notably, SNPs mapping on BTA 23 largely captured most of the genetic variation for both traits, influencing BLV levels of infection. Overall, a group of 24 SNPs were significantly associated with high PVL, whilst 11 SNPs were associated to high WBCs counts. Of these, only 1 SNP (rs110277740) is not shared with those associated with PVL (Table 1). PVL and WBCs are correlated (~ 0.81 (SE ± 0.10)). Joint significant positions for PVL and WBCs taken together delimit a region on BTA 23 that is located within the bovine MHC, approximately from the beginning of bovine leukocyte antigen (BoLA) class IIa region (~ 20.6 Mb) extending to the Histone cluster I which mark off the end of the complex (~ 33.3 Mb). Only a small fraction of phenotypic variance is explained by SNPs located outside this MHC region, so we decided to repeat the GWAS for PVL but excluding the SNPs located between ~ 20–36 Mb of BTA 23, which could be masking the SNPs effects in non-MHC regions. As result, we did not find any significantly associated SNP (Additional file 5: Table S1).

**Table 1:** Significantly associated variants in the GWA studies based on MLMs

refSNP_ID	BTA	PVL			WBC			Position ^c^
		b ^a^	SD	p ^b^	b ^a^	SD	p ^b^	
rs110155623	23	−0.5	0.10	7.20 × 10 ^− 07^	−0.44	0.09	6.23 × 10 ^− 07^	20,692,320
rs41587216	23	−0.15	0.03	5.06 × 10 ^− 07^	−0.13	0.03	4.95 × 10 ^− 07^	22,300,959
rs41566363	23	−0.18	0.03	1.97 × 10 ^− 07^	−0.15	0.03	5.53 × 10 ^− 07^	22,997,898
rs41641297	23	0.21	0.03	2.80 × 10 ^−10^	0.15	0.03	5.19 × 10 ^− 08^	24,181,053
rs109343703	23	0.24	0.04	1.95 × 10 ^− 08^	–	–	–	24,699,202
rs110499907	23	0.21	0.04	1.96 × 10 ^− 07^	–	–	–	24,727,613
rs110473048	23	0.21	0.03	1.78 × 10 ^−12^	0.14	0.03	9.97 × 10 ^−08^	25,109,188
rs110525467	23	−0.36	0.04	5.32 × 10 ^−16^	−0.24	0.04	5.90 × 10 ^−10^	25,426,985
rs110579760	23	0.25	0.04	3.66 × 10 ^−09^	–	–	–	25,507,676
rs110836188	23	0.23	0.04	6.50 × 10 ^−11^	–	–	–	27,088,825
rs41255514	23	0.23	0.03	1.47 × 10 ^−11^	–	–	–	27,305,227
rs17872223	23	0.22	0.03	6.99 × 10 ^−12^	–	–	–	27,306,795
rs110861313	23	0.19	0.03	7.02 × 10 ^−11^	0.12	0.02	5.70 × 10 ^−07^	27,444,064
rs17871874	23	0.17	0.03	2.69 × 10 ^−07^	0.15	0.03	1.96 × 10 ^− 07^	27,485,467
rs110260956	23	0.21	0.04	7.70 × 10 ^−07^	–	–	–	27,545,231
rs110350951	23	0.14	0.03	1.54 × 10 ^−07^	–	–	–	27,841,983
rs110794231	23	−0.16	0.03	6.44 × 10 ^− 09^	–	–	–	27,887,914
rs41587536	23	−0.15	0.03	2.25 × 10 ^− 08^	–	–	–	27,923,154
rs110742604	23	−0.26	0.03	6.67 × 10 ^−14^	−0.16	0.03	8.18 × 10 ^− 08^	28,087,630
rs109856572	23	−0.18	0.03	1.27 × 10 ^− 08^	−0.14	0.03	1.72 × 10 ^− 07^	28,649,349
rs110769723	23	−0.25	0.04	3.01 × 10 ^− 08^	–	–	–	29,285,952
rs109015676	23	−0.21	0.04	4.82 × 10 ^− 07^	–	–	–	29,535,762
rs110034224	23	−0.18	0.04	5.46 × 10 ^− 07^	–	–	–	30,662,593
rs110277740	23	–	–	–	−0.19	0.03	2.20 × 10 ^− 08^	31,286,064
rs109754326	23	−0.15	0.03	2.99 × 10 ^− 07^	–	–	–	33,296,630

*Abbreviations*: refSNP_ID: SNP Identification based on dbSNP (http://www.ncbi.nlm.nih.gov/SNP/), PVL: proviral load, WBC: whole blood cells counts, SD: standard deviation

^a^SNP effect size (β of regression)

^b^*p*-value: Under null hypothesis, probability of obtaining the observed effect size

^c^SNP location according to bovine genome assembly UMD3.1

Unless a significant SNP is itself the causal variant, it will capture the effects of other variants in LD responsible for the phenotype. The clustering of genes with related functions in the linkage disequilibrium pattern in the bovine MHC region makes it difficult to detect effects of individual *loci*. We characterized the LD extent (Additional file 6: Figure S5) in our Holstein and Holstein x Jersey population. At distances ≤30 kb, the average correlation value r^2^ was 0.26 ± 0.30 (34% of pairwise SNPs showed r^2^ > 0.25). This value rapidly decays to 0.14 ± 0.21 when the inter-markers distance was between 50−100 kb. About 60% of the inter-SNPs distances in this work is in the range of 25–50 kb with an average r^2^ = 0.22 ± 0.27, indicating that causal genetics variants effects could be retrieved from SNPs hits.

**Fig. 4:**
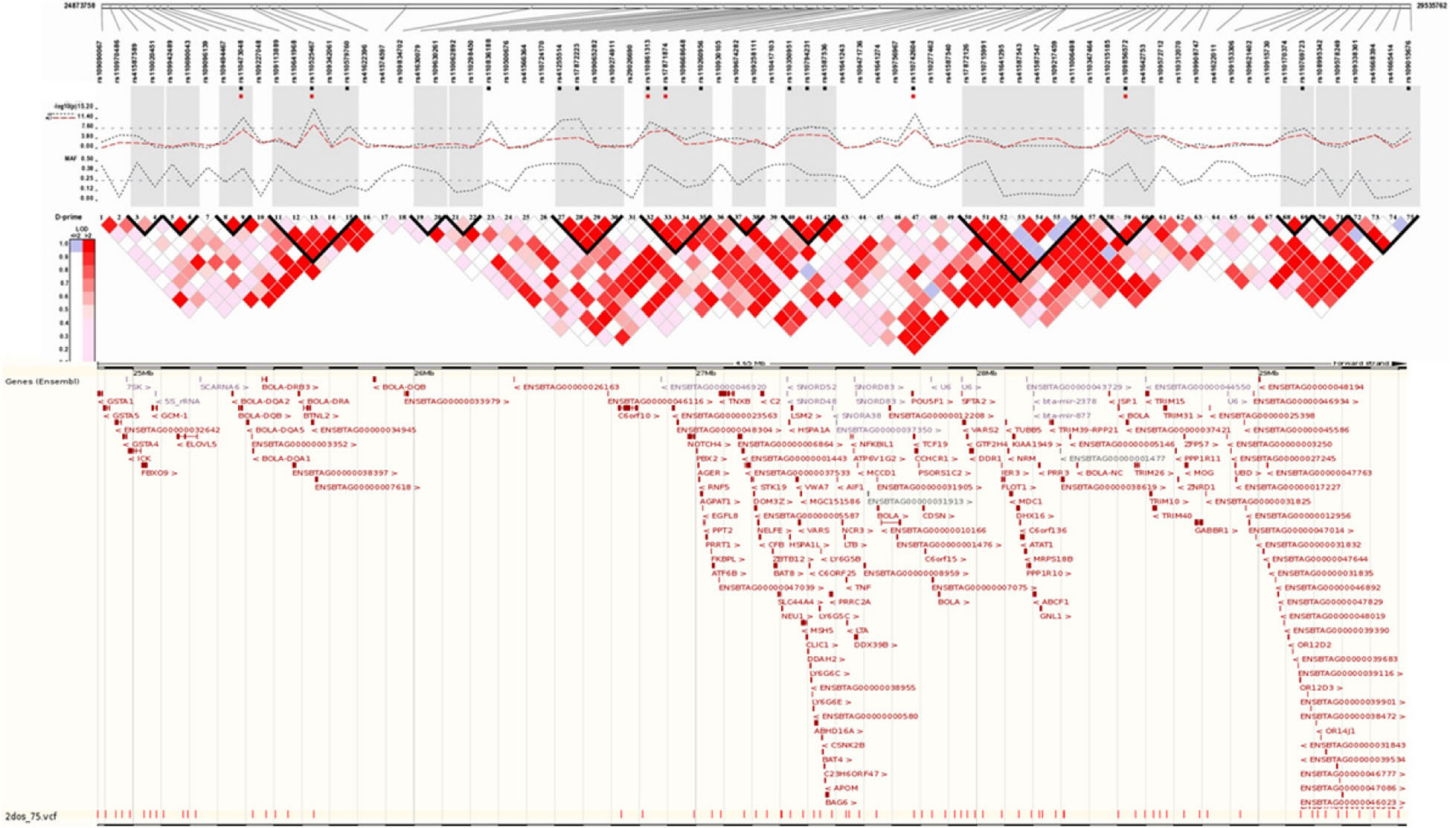
Gene context and LD map of the bovine chromosome 23 containing the SNPs significantly associated in the GWAS. A) BTA23:24,872,758–29,535,762 bp. ■ PVL significant SNPs; red square symbol: WBCs significant SNPs; ----- log_10_(p)_PVL; red broken line: log_10_(p)_WBC; MAF: Minimum Allele Frequency. Local LD map estimates (D´) based on 235 SNPs located within the cattle MHC region in founder maternal chromosomes. Triangles delimited with black lines identify haplotype blocks derived from Haploview [55]. The gene content of the region according to the bovine genome assembly UMD3.1 was obtained from Ensembl Genome Browser (http://www.ensembl.org). Red vertical bars indicate SNPs of the SNP50K chip located in the region

We found five significantly associated SNPs pairs having a strong allelic correlation (r^2^ > 0.80). The rs110579760 (BTA23: 25,507,676 bp) was in LD with rs109343703 (r^2^ = 0.95; BTA23: 24,699,202 bp) and rs110499907 (r^2^ = 0.88; BTA23: 24,727,613 bp). The r^2^ between rs109343703 and rs110499907 was 0.90. The remaining pairs were rs41255514 (BTA23: 27,305,227 bp) and rs17872223 (BTA23: 27,306,795 bp) with r^2^ = 0.96, and the pair rs110794231 (BTA23: 27,887,914 bp) and rs41587536 (BTA23: 27,923,154 bp) had the same r^2^ value. Figure 4 shows the gene context and the LD map in the BTA23: 24,872,758–29,535,762 bp region.

SNPs were assigned to putative biological function if they fell within a coding, intronic, or regulatory region. For those SNPs mapping outside genic regions we scrutinised the closest gene located at a distance where a moderate LD was kept. A total of 44 genes were identified using the criteria described above. Table 2 details the genomic context of each SNP (position and related genes). Half of significant SNPs mapped to putative regulatory regions, 24% to intergenic regions, 12% to introns and 12% of SNPs to exons. Only one SNP (rs41255514) is located in a 3′-untranslated region (3’-UTR). The SNP with the lowest *p*-value for both conditions was rs110525467 (p_PVL_ = 5.32 × 10^− 16^ and p_WBC_ = 5.90 × 10^− 10^) introducing a synonymous substitution in the MHC class II gene *BoLA-DQA1* (ENSBTAG00000037605) located in BTA23: 25,426,330–25,430,097 bp. The remaining 10 positions associated with a high number of peripheral blood leukocyte counts included an intronic SNP located in the *Polycystic kidney and hepatic disease 1* gen, (*PKHD1* - ENSBTAG00000011237) and a synonymous substitution in the *Proline-rich coiled-coil 2A* gene (*PRRC2A* - ENSBTAG00000019682). In addition, six SNPs significantly associated with this condition were located in regulatory regions of the *GPR111* (ENSBTAG00000037687), *CD2AP* (ENSBTAG00000000322), *TFAP2D* (ENSBTAG00000020425), *C23H6ORF47* (ENSBTAG00000023628), *ABHD16A* (ENSBTAG00000000578), *LY6G5C* (ENSBTAG00000025449), *LY6G5B* (ENSBTAG00000039740), *CSNK2B* (ENSBTAG00000008837), *BAT4* (ENSBTAG00000008835), *APOM* (ENSBTAG00000008833), *BAG6* (ENSBTAG00000019685), *TUBB5* (ENSBTAG00000006969), *IER3* (ENSBTAG00000011358), *FLOT1* (ENSBTAG00000009960), *TRIM31* (ENSBTAG00000004490), *TRIM40* (ENSBTAG00000037381) and *ABT1* (ENSBTAG00000010784) genes (Table 2). The two remaining SNPs, rs41587216 and rs110473048, were at 39.3 kb of *CRISP1* (ENSBTAG00000008085) and 21.7 kb *GCM-1* (ENSBTAG00000008148), respectively.

**Table 2:** Genes linked to SNPs identified in the GWA analysis for PVL and WBCs

refSNP_ID	Position ^a^	A1 ^b^	MAF	Variant type ^c^	Symbol ^d^	Description
rs110155623	20,692,320	A	0.03	Regulatory intergenic	*GPR111, CD2AP*	*G protein-coupled receptor 111; CD2-associated protein*
rs41587216	22,300,959	A	0.46	Intergenic	*CRISP1*	*Cysteine-rich secretory protein 1*
rs41566363	22,997,898	C	0.33	Regulatory intergenic	*TFAP2D*	*Transcription factor AP-2 delta (activating enhancer binding protein 2 delta)*
rs41641297	24,181,053	A	0.40	Intronic	*PKDH1*	*Polycystic kidney and hepatic disease 1*
rs109343703	24,699,202	A	0.12	Intronic	*TRAM2*	*Translocation associated membrane protein 2*
rs110499907	24,727,613	A	0.14	Intergenic	*TRAM2*	*Translocation associated membrane protein 2*
rs110473048	25,109,188	A	0.34	Intergenic	*GCM-1*	*Glial cells missing homolog 1*
rs110525467	25,426,985	G	0.19	Synonymous substitution	*BOLA-DQA1*	*Major histocompatibility complex, class II, DQA1*
rs110579760	25,507,676	G	0.12	Intergenic	*BOLA-DRB3*	*Major histocompatibility complex, class II, DRB3*
rs110836188	27,088,825	G	0.21	Synonymous substitution	*TNXB*	*Tenascin-X precursor*
rs41255514	27,305,227	G	0.34	3′-UTR	*SCL44A4*	*Solute carrier family 44, member 4*
rs17872223	27,306,795	G	0.33	Intronic	*NEU1*	*Sialidase 1 (lysosomal sialidase)*
rs110861313	27,444,064	C	0.46	Regulatory intergenic	*C23H6ORF47, ABHD16A, LY6G5C, LY6G5B, CSNK2B, BAT4, APOM, BAG6*	*Chromosome 23 open reading frame, human C6orf47; Abhydrolase domain containing 16A; Lymphocyte antigen 6 complex, locus G5C; Lymphocyte antigen 6 complex, locus G5B; Casein kinase 2, beta polypeptide; G patch domain and ankyrin repeats 1; Apolipoprotein M; BCL2-associated athanogene 6*
rs17871874	27,485,467	A	0.24	Synonymous substitution	*PRRC2A*	*Proline-rich coiled-coil 2A*
rs110260956	27,545,231	G	0.15	Regulatory intergenic	*LTA, NFKBIL1, ATP6V1G2,LTB,TNF*	*Lymphotoxin alpha; Nuclear factor of kappa light polypeptide gene enhancer in B-cells inhibitor-like 1; ATPase, H+ transporting, Lysosomal 13 kDa, V1 subunit G2; Lymphotoxin beta; Tumor necrosis factor*
rs110350951	27,841,983	G	0.44	Regulatory intergenic	*U6, Ig_like_MHC_I*	*U6 small nuclear RNA; Major histocompatibility complex, class I, A-like*
rs110794231	27,887,914	A	0.38	Intergenic	*BOLA*	*Bola class I*
rs41587536	27,923,154	G	0.38	Intergenic	*SFTA2*	*Surfactant associated 2*
rs110742604	28,087,630	G	0.20	Regulatory intergenic	*TUBB5, IER3, FLOT1*	*Tubulin, beta class I; Immediate early response 3; Flotillin 1*
rs109856572	28,649,349	A	0.41	Regulatory intergenic	*TRIM31, TRIM40*	*Tripartite motif containing 31; Tripartite motif containing 40*
rs110769723	29,285,952	G	0.18	Regulatory intergenic	*LOC516273, LOC618064, LOC785162*	*Olfactory receptor; Olfactory receptor; Olfactory receptor*
rs109015676	29,535,762	G	0.12	Regulatory intergenic	*LOC784787,LOC511103, LOC784858*	*Olfactory receptor; Olfactory receptor; Olfactory receptor*
rs110034224	30,662,593	C	0.17	Regulatory intergenic	*LOC522315*	*Olfactory receptor*
rs110277740	31,286,064	G	0.17	Regulatory intergenic	*ABT1*	*Activator of basal transcription 1*
rs109754326	33,296,630	G	0.34	Regulatory intergenic	*NRSN1*	*Neurensin 1*

*Abbreviations*: refSNP_ID: dbSNP SNPs Identification (http://www.ncbi.nlm.nih.gov/SNP/), MAF: Minor Allelic Frequency, NA: Not Applicable

^a^SNP location according to bovine genome assembly UMD3.1

^b^Allele with the lowest frequency in the 50KSNP chip according to the Illumina nomenclature TOP/BOT

^c^Intergenic: SNP positioned between 20 and 55 kb to the nearest gene; Regulatory intergenic: SNP positioned 0-20Kb upstream or downstream of a gene; Synonymous substitution: exonic SNP producing a synonymous amino acid substitution in the gene; 3’-UTR: SNP positioned in the 3′ untranslated region of a gene; Intronic: SNP positioned in gene introns

^d^Gene symbol according to UniProtKB (http://www.uniprot.org/)

**Fig. 5:**
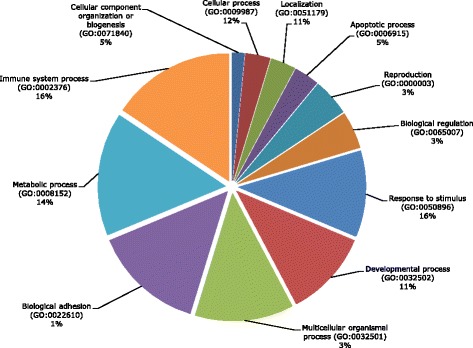
Distribution of the GO “Biological Process” (BP) categories for genes represented by the significantly associated SNPs in GWAS for PVL and WBC counts

The PVL phenotype association study included all significant SNPs for the GWAS considering WBCs -except the SNP assigned to *Activator of Transcription Basal 1* gene (*ABT1*, rs110277740) - and other 14 significant SNPs. The functional annotation resulted in a synonymous substitution in *TNXB* (ENSBTAG00000001444) and a SNP located in the 3’-UTR region of *SCL44A4* (ENSBTAG00000005675). Also, there are two SNPs located in intronic regions of *NEU1* and *TRAM2* (ENSBTAG00000009112 and ENSBTAG00000005674, respectively). The variant rs110260956 is 20 kb upstream or downstream of the following genes: *LTA* (ENSBTAG00000000016), *NFKBIL1* (ENSBTAG00000014492), ATP6V1G2 (ENSBTAG00000014491), *LTB* (ENSBTAG00000020674) and *TNF* (ENSBTAG00000025471), while rs110350951 meets the same condition in *U6 snRNA* (ENSBTAG00000043004) and *Ig-like MHCI* genes (ENSBTAG00000007075), and rs109754326 in *NRSN1*gene (ENSBTAG00000009180). Three SNPs significantly associated with high PVL are present in putative regulatory regions of 7 genes encoding Odorant receptors (Olfactory receptor). Finally, rs110579760, rs110794231 and rs41587536 polymorphisms are at 30.7, 20.3 and 22.3 kb of *BoLA-DRB3* (ENSBTAG00000013919) *BoLA-A* (ENSBTAG00000022590) and *SFTA2* (ENSBTAG00000038810), respectively. Genes represented by the SNPs were assigned to Gene Ontology (GO) functional terms and the PANTHER (Protein Class, PC) (http://pantherdb.org/) classification system. The analysis was performed for all genes represented by significantly associated SNPs with any of the two traits. A total of 11 PC “parents” terms were identified being the three most common categories “protein defense/immunity”, (PC00090) (21,7%), “signalling molecule” (PC00207) (21,7%) and cytoskeletal protein (PC00085) (8,7%). In Additional file 7: Table S2 a description of each PC term is displayed along with their “child” terms providing specificity to the functional description. Notably, a single gene might be contained in more than one PC class. Afterwards, we classified genes under the category GO “Biological Processes” (BP) wherein each gene identification is assigned according to their function within a network of proteins that collectively carry out a particular process within the cell. Figure 5 presents the distribution of 12 “parent” terms found according to GO BP classification, these represent general cellular processes being the top five terms “immune system process” (16%), “response to stimulus” (16%), “metabolic process” (14%), “cellular process” (12%) and “localization” (11%). The search for GO terms and/or KEGG pathways overrepresented on the gene set identified vs those present in whole bovine gene set using FatiGO method [68] did not produce significant results (data not shown).

## Discussion

The humoral immune response plays a crucial role in the control of viral infections in cattle and humans, and the level of response varies from individual to individual, being this variability highly heritable [69, 70]. In cattle, we showed that the humoral response to BLV infections indeed varies among animals and, interestingly, it can be used to infer the circulating PVL in an infected animal [8]. In this study, we use the % of Reactivity (anti-p24) to screen a population of BLV naturally infected animals aiming to select cows most likely to have extreme values ​​of PVL set-point, which was subsequently confirmed by qPCR. The predictive ability of serological markers to detect high PVL conditions was good being the ROC area under the curve > 0.80 (data not shown). We then performed a case-control study setting levels of infection (LI) as phenotype aiming to estimate (a) the proportion of phenotypic variation that can be attributed to genetic components and (b) the cattle genome mapping positions of genetic polymorphisms underlying this variation. A positive genetic correlation between PVL and WBCs of magnitude ~ 0.81 (SE ± 0.10) supports the idea of shared or genomic regions influencing both traits.

We used kinship captured from QC pruning variants to assess the additive genetic variance of LI produced by BLV in the population. The percentage of variation reached was 63% (h^2^_GRM_PVL_ = 0.63) and 56% (h^2^_GRM_WBC_ = 0.56) on the risk scale or liability. On this scale, the phenotypic variances do not depend on the disease prevalence, so h^2^_lia_ values allow comparisons between populations and environments where incidence varies. When partitioned, the proportion of the phenotypic variance explained by markers for both phenotypes was distributed on different chromosomes. In a pedigree-based study by Abdalla et al. [71], the contribution of genetic components to the incidence of BLV in Holsteins and Jersey herds (approximately 30% of herd level prevalence) was estimated to be 8%, showing that environmental factors could play a major role over genetics in susceptibility to BLV occurrence. Paradoxically, the implementation of good veterinary practices and proper management in order to reduce iatrogenic issues, considered one of the main factors of infection among cows within herds [44], failed to reduce the prevalence ([7, 43]). However, we focused on phenotypes representing the BLV level of infection in cows. The LI is directly related to the cows’ viral transmission ability to BLV free herd mates. Methods incorporating GRM as used in this study, have been successfully tested in selective breeding of animals and plants for complex traits [72, 73] and proposed to predict human disease phenotypes [74–76]. The high heritability values obtained for LI have remarkable practical implications in genetic improvement programs aiming to reduce the dissemination of BLV in heavily infected populations. Sources of non-additive genetic variance, such as dominance or epistasis among markers, were not considered in this study; however, the evidence indicates that the additive genetic variance is the major contributor to the phenotypic variance in complex characters [77–79]. To our knowledge, this is the first report that estimates the level of phenotypic variation in PVL and WBC explained by SNPs markers in BLV infected animals.

The molecular basis influencing the inter-individual difference in LI in vivo during aleukemic phase remains unexplained. The mechanisms of BLV infection involve an infectious cycle after a primary infection, where the virus replicates and spreads within the host [80] by cell-to-cell virion transference, but this process is promptly abrogated by the anti-BLV host immune response [81]. Subsequently, the PVL is maintained by polyclonal expansion of surviving infected B-cells elapsed by mitotic division where viral factors hijack host cellular physiological pathways.

To evaluate which genes might be influencing the set-point of PVL and WBC counts, we identified genomic regions harbouring significantly associated SNPs (*p* < 1.13 × 10^− 6^). These SNPs were located in the MHC region on BTA 23, the most relevant gene cluster of the immune sub-genome with high gene density and exceptional diversity.

Polymorphisms in the MHC *loci* −featured by an intricate structure of linkage disequilibrium [82–85]− are associated with viral infections outcomes, such as HIV-1, HBV, HCV and HPV [86–88]. Fellay et al. [89, 90] confirmed through GWAS that polymorphisms in HLA are leading host genetic determinants on the viral load set-point in HIV−1 infected individuals. To investigate whether effects in others *loci* were masked by the BoLA region, we have also performed the same genome-wide analysis with the PVL level but excluding SNPs within the bovine MHC. No tested SNP reached significance level (Additional file 5: Table S1). However, when BLV incidence (based on ELISA test) was considered as phenotype for GWAS in US Holstein and Poland Holstein-Frisian populations, many genes outside of the MHC appears to be modulating this trait [40, 41]. The phenotype evaluated in these studies (BLV-seronegatives vs BLV-seropositives cows) indicated novel potentially associated genomic regions, however they do not corroborate previous finding suggesting the MHC as the main genomic region harbouring genes influencing BLV infection [28, 31, 33].

Genomic positions highlighted by GWAS emphasised the potential influence of polymorphic variants in MHC class I and II genes related to their central role in cell-mediated immunity. Four SNPs shared the functional term *antigen processing and presentation* (GO:0019882). The SNP with the lowest *p*-value ​​for both conditions studied was rs110525467 (p_PVL_ = 5.32 × 10^− 16^ and p_WBC_ = 5.90 × 10^− 10^) producing a synonymous substitution in the MHC class II gene *BoLA-DQA1.* The rs110579760 (p_PVL_ = 3.66 × 10^− 09^) located 30.7 kb downstream *BoLA-DRB3* was significantly associated with PVL, but not with WBC (Table 1). After infection, a strong cytotoxic T-cell response is developed in peripheral blood against specific viral epitopes [91]. In this regard, two significantly associated SNPs in strong LD (r^2^ = 0.96) were functionally related with *BoLA−A* and *SFTA2* (Surfactant Associated 2) genes. The magnitude of the immune response to viruses exerted by CD8+ T cells and its association with polymorphisms in HLA class I molecules is widely described in the literature [92–94]. In the case of BLV infections in cattle, previous studies have identified associations between polymorphisms in MHC class I genes and resistance to PL in different breeds [26, 27, 95, 96]. The closest SNP (rs110794231) to *BoLA-A* is 35.2 Kb away corresponding to the *SFTA2* gene. The *SFTA2* gene encodes a secreted hydrophilic protein with surfactant properties with a role in host defence pathogen recognition patterns (bacteria, viruses and fungi) [97, 98].

The SNP rs41587216 (p_PVL_ = 5.06 × 10^− 07^ and p_WBC_ = 4.95 × 10^− 07^) was positioned in the secreted protein CRISP1 (Cysteine-rich secretory protein 1), expressed in skeletal muscle, salivary/lacrimal glands and during lymphoblast development [99] potentially interfering in innate and adaptive immunity (GO:0002376 “*immune system process*”). Other GWAS hit, the SNP rs109856572, is involved in biological processes that are associated with anti-retroviral innate immunity. It is located in a transcriptional regulatory region of *TRIM31* and *TRIM40* genes that pertain to the E3 ubiquitin ligases protein family [100–103]. Perhaps the most studied TRIM mechanism is the restriction of infections caused by HIV−1 exerted by TRIM5α [104–106].

A particular haplotype block located in the MHC Class III region, encompasses a number of genes with a plethora of biological effects that may explain the associations detected (Fig. 4). These genes play relevant roles in the immune response, specifically in the regulation of apoptosis and proteasome degradation (*LTA, TNF, BAG6)* [107], HIV−1 progression (*BAT1–5, LY6*) [86], T cells response to interferon and viral shedding prevention during innate response to infections (*LY6*) [108, 109].

Tumor necrosis factor (*TNF*) and LTA are pro-inflammatory cytokines classified to the GO BP “*apoptotic process*” (GO:0006915). Polymorphisms in TNF have been reported to contribute partially to the development of lymphosarcoma and PVL in BLV infected cattle [30, 110] and early elimination of BLV in experimentally infected sheep [111].

Several of the targeted genomic regions in the GWAS might be involved in viral intra-host spreading after primary infection according to mechanisms proposed in [81]. For example, in the case of cells-cells fusions the SNP rs110473048 (p_PVL_ = 1.78 × 10^− 12^ and p_WBC_ = 9.97 × 10^− 08^) is at 7.21 kb from the Transcription Factor (TF) *GCM1* (*Glial Cells Missing 1*) gene that regulates the expression of the *ENV* gene (located in the endogenous retrovirus HERV-W) [112] encoding for a fusogenic protein called Syncytin-1. Recently, it has been postulated that infection by an exogenous virus can *trans*˗activate *ENV* ectopically by increasing the expression of GCM1 [113] and promoting syncytia formation in breast cancer and endometrial carcinoma [114, 115].

Two significantly associated SNPs mapped within and close to *TRAM2* (Translocation-Associated Membrane Protein *2*) gene, involved in the biosynthesis and excretion of type I collagen [116] that constitutes the extracellular matrix components [117] where virions are stored. The SNP rs109343703 causes an intronic substitution and the rs110499907 SNP is located 24.7 kb upstream of it. Both SNPs were associated with PVL and not with WBCs, and the nearest SNP in *BoLA-DRB3* was in strong LD (r^2^ > 0.85) with them. Another associated polymorphism (rs110836188) produces a synonymous substitution in the *TNXB* gene (Tenascin-X), which encodes an extracellular matrix protein involved in matrix-cell adhesion (GO:0007160) and organization of collagen fibres (GO:0030199). Ultimately, analogous to “immunological synapses” some viruses subvert cellular processes to disseminate in the host through “virological synapses” [118, 119]. In this process, cytoskeletal proteins are required for polarization of cells involved in the synapses [120, 121]. Crucially, the SNP rs110155623 associated with both phenotypes is located in the putative regulatory region of *CD2AP* (CD2-associated protein). CD2AP organizes the cytoskeleton in polarization sites (GO:2,000,249) interacting directly with the Actin protein [122]. Two other GWAS hit variants could capture the effects of genes related to regulation and polymerization of the cytoskeleton structures. A substitution in an intron in the polycystic kidney and hepatic disease 1 (*PKHD1*) gene (rs41641297) and a SNP (rs110742604) positioned in regulatory regions of *TUBB5* and *FLOT1* genes. The *TUBB5* gene encodes β5-tubulin which belongs to the *β*-tubulin family.

Finally, four SNPs were assigned to TFs (Table 2) regulating transcriptional processes in promoters depending on RNA polymerase II (GO:0006357). TF genes are candidates for host determinants controlling BLV intra−host spreading, due to a tight mechanism of transcriptional regulation exerted by the *trans*-activating protein Tax, modulating several host signalling pathways such as AP-1, NF-κB and CREB to induce oncogenic transformation [123].

Altogether, in addition to classical immune response related genes, in this study we pointed novel genes/mechanisms as possibly involved in PVL control and hence BLV dissemination.

Despite our results do not conclusively identify causal genomic variants; we have successfully contracted the landscape of genes potentially related to BLV infections to a short list of very specific candidate genes suitable for experimental validations by gene-expression assays. These results also encourage fine mapping and validation studies. Furthermore, functional studies integrating multi-omics data will allow discerning the underlying molecular mechanisms of the BLV−host interaction.

## Conclusions

Results obtained in this study revealed relevant genetic variants in the bovine MHC strongly influencing the control of BLV infection in cattle. Functional inspection of genes harbouring statistically significant associated SNPs brought preliminary evidence about possible biological pathways underlying the LI in infected cows. The proviral BLV load is associated with the stage of the disease progression in the infected cows and directly determines its ability to transmit BLV to healthy animals. Therefore, genome wide identification of genetics variants associated with low proviral load BLV infections would be useful not only to design therapeutics and preventive treatments but also as alternative control programs based on selective breeding of animals through genomic selection in dairy cattle.

## Additional files

Additional file 1: **Figure S1.** Pedigree of the Holstein and Jersey crosses cattle population under study. Red lines connect the individual with his father. Blue lines connect the individual with his mother. (DOCX 252 kb).

Additional file 2: **Figure S2**. Holstein and Jersey crosses population sub-structure. Each point represents the coordinates of PC1 and PC2 for each individual. The assignment of PCs allowed inferences about the ancestry origin of cows. (0; 12.5; 25; 50; 75 and 100 of Holstein breed percentage). (DOCX 236 kb).

Additional file 3: **Figure S3.** QQ plots and genome inflation factor (λ) in GWAS with PVL. The *p*-values come from a logistic regression under an additive model of association of SNPs with the level of PVL. The genomic inflation factor quantifies the volume of inflation observed. A) A strong deviation in the -log_10_(p) (black circle) from the line of identity (red line) was observed without considering covariates, λ = 5.31. B) A small adjustment was observed when included the following covariates: Age of the animal (A), Herd (H), Number of lactation (L) Percentage of Holstein (PH) and Bull (B), λ = 4.07. C) A significant correction is manifested including the first 8 Principal Components (PC1-PC8), λ = 1.26. D) When considering all the covariates together, the correction level approaches the CI95% of the null distribution (dotted lines), λ = 1.21. (DOCX 404 kb).

Additional file 4: **Figure S4**. QQ plots and genome inflation factor (λ) in GWAS with WBC counts. Analyses were based on the -log_10_(p) from logistic regression under an additive model of association of SNPs with WBCs. A) A pronounced deviation from the null distribution (red line) is manifested in the model without covariates, λ = 3.99. B) When covariates A_DF_L_PH_B were adjusted in the model λ decreases to 2.76, C) A significant correction is achieved by considering PC1-PC8, λ = 1.31. D) When considering all the covariates together (PC1-PC8_A_H_L_PH_B) the correction level approached the CI 95% of the null distribution (dotted lines), λ = 1.30. (DOCX 429 kb)

Additional file 5: **Table S1.** Top 15 SNPs with the lowers *p*-value from the GWA analysis with PVL excluding SNPs located ~ 20–36 Mb of BTA 23 (DOCX 20 kb).

Additional file 6: **Figure S5.** LD decay by distance. LD measures were considered among syntenic SNPs pairs separated < 1 Mb. The r^2^ values were averaged each 10 Kb and plotted versus inter-SNPs distances. (DOCX 56 kb).

Additional file 7: **Table S2.** Description of PC categories identified and genes containing them. (DOCX 22 kb).

## Abbreviations

BLV: Bovine Leukemia Virus
BoLA: Bovine leukocyte antigen
BTA: *Bos taurus* autosome
ELISA: Enzyme-linked Immunosorbent Assay
GO: Gene ontology terms
GRM: Genetic relationship matrix
GWAS: Genome-Wide Association Study
HLI: High level of infection
HPVL: High proviral load
IBS: Identity by State
LD: Linkage Disequilibrium
LMMs: Linear Mixed Models
LPVL: Low proviral load
MAF: Minor Allele Frequency
MHC: Major Histocompatibility Complex
PBMCs: Peripheral Blood Mononuclear Cells
PCs: Principal Components
PL: Persistent Lymphocytosis
PVL: Proviral Load
QC: Quality Control
SNPs: Single Nucleotide Polymorphisms
WBC: White Blood Cells
