# Supplementary figures and images for: Genome-wide scan for commons SNPs affecting bovine leukemia virus infection level in dairy cattle
Source: BMC Genomics. 2018 Feb 13;19:142. doi: 10.1186/s12864-018-4523-2 (PMC5812220; doi:10.1186/s12864-018-4523-2)

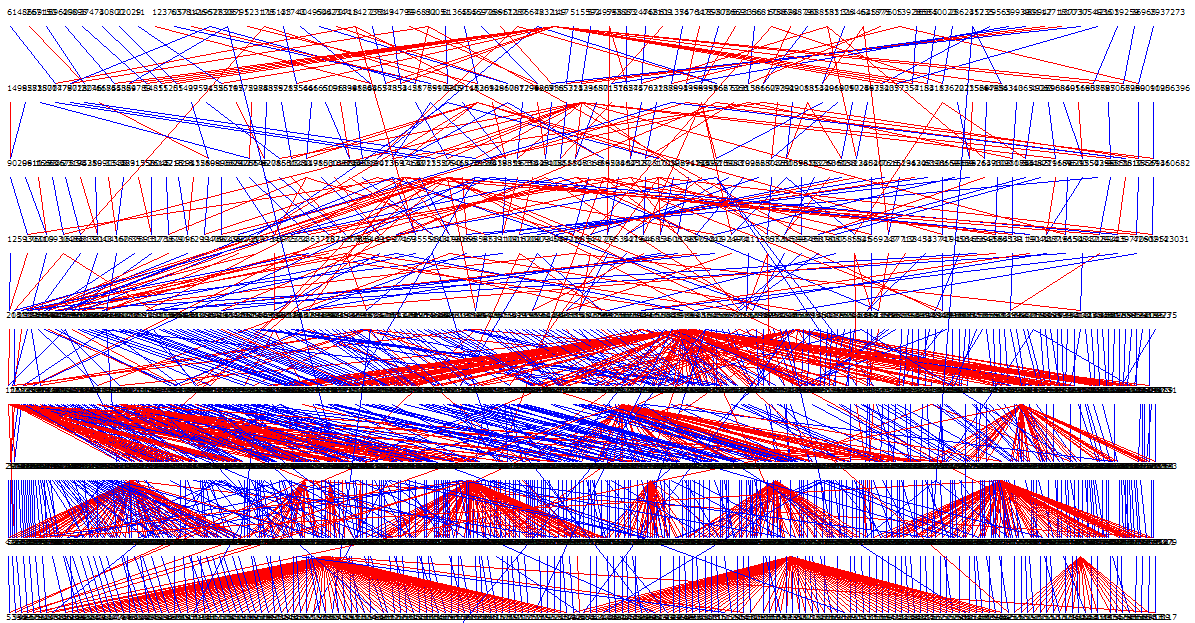

Supplement: Supplementary file 1 — Figure S1. Pedigree of the Holstein and Jersey crosses cattle population under study. Red lines connect the individual with his father. Blue lines connect the individual with his mother. (DOCX 252 kb). [file 12864_2018_4523_MOESM1_ESM.docx]

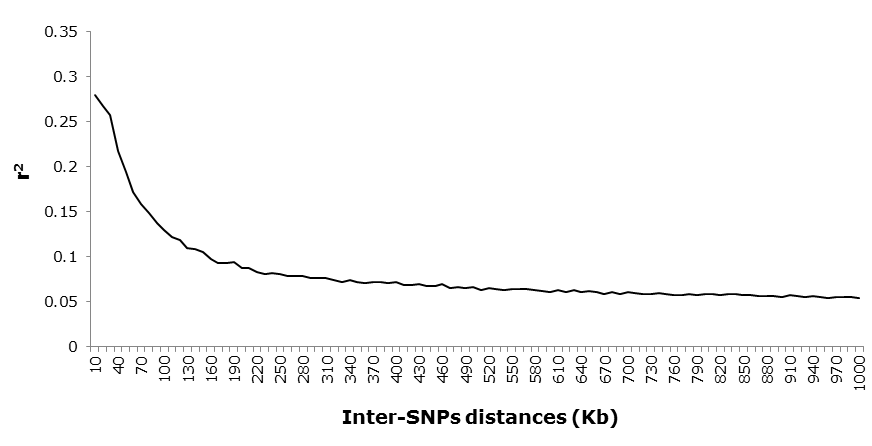


r2

Inter-SNPs distances (kb)

Supplement: Supplementary file 6 — Figure S5. LD decay by distance. LD measures were considered among syntenic SNPs pairs separated < 1 Mb. The r2 values were averaged each 10 Kb and plotted versus inter-SNPs distances. (DOCX 56 kb). [file 12864_2018_4523_MOESM6_ESM.docx]
