# Supplementary material for: Genome-wide scan for commons SNPs affecting bovine leukemia virus infection level in dairy cattle
Source: BMC Genomics. 2018 Feb 13;19:142. doi: 10.1186/s12864-018-4523-2 (PMC5812220; doi:10.1186/s12864-018-4523-2)
Supplement: Supplementary file 3 — Figure S3. QQ plots and genome inflation factor (λ) in GWAS with PVL. The p-values come from a logistic regression under an additive model of association of SNPs with the level of PVL. The genomic inflation factor quantifies the volume of inflation observed. A) A strong deviation in the -log10(p) (black circle) from the line of identity (red line) was observed without considering covariates, λ = 5.31. B) A small adjustment was observed when included the following covariates: Age of the animal (A), Herd (H), Number of lactation (L) Percentage of Holstein (PH) and Bull (B), λ = 4.07. C) A significant correction is manifested including the first 8 Principal Components (PC1-PC8), λ = 1.26. D) When considering all the covariates together, the correction level approaches the CI95% of the null distribution (dotted lines), λ = 1.21. (DOCX 404 kb). [file 12864_2018_4523_MOESM3_ESM.docx]

Expected –log_10_(p)

Observed –log_10_(p)

Observed –log_10_(p)

Observed –log_10_(p)

A) λ= 5.31.

C) PC1-PC8 λ= 1.26.

B) A_H_L_PH_B λ= 4.07.

D) PC1-PC8_A_H_L_PH_B λ= 1.21.


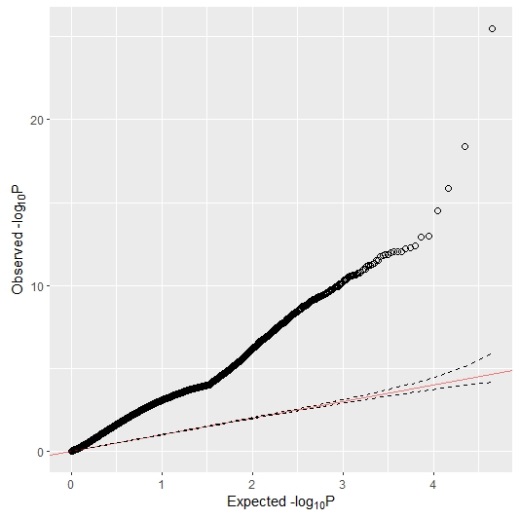

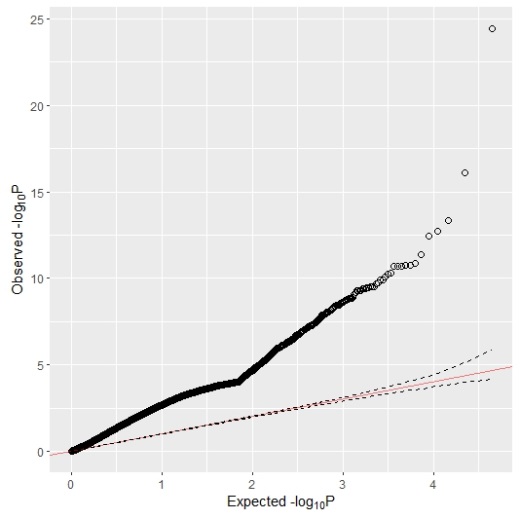

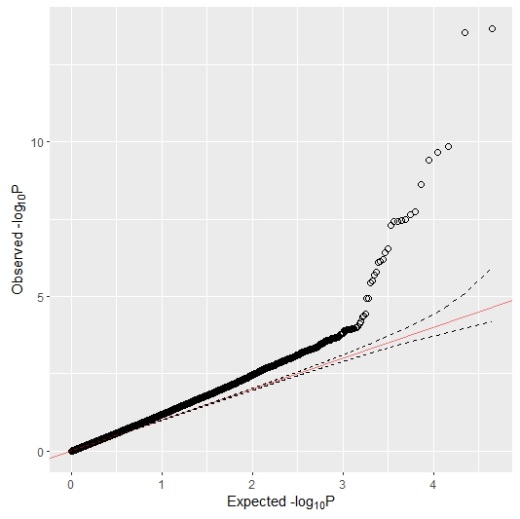

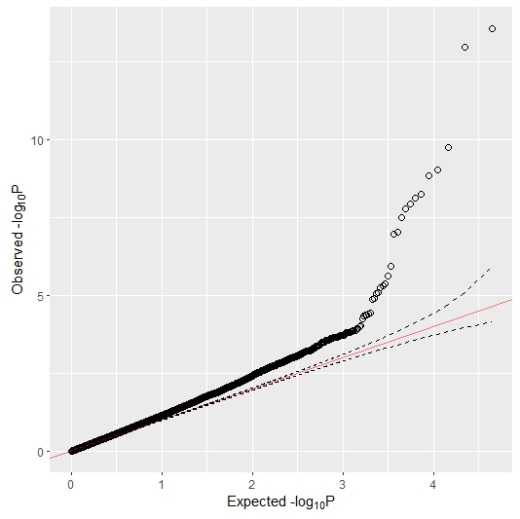


Observed –log_10_(p)

Expected –log_10_(p)

Expected –log_10_(p)

Expected –log_10_(p)
