# Supplementary material for: Genome-wide scan for commons SNPs affecting bovine leukemia virus infection level in dairy cattle
Source: BMC Genomics. 2018 Feb 13;19:142. doi: 10.1186/s12864-018-4523-2 (PMC5812220; doi:10.1186/s12864-018-4523-2)
Supplement: Supplementary file 4 — Figure S4. QQ plots and genome inflation factor (λ) in GWAS with WBC counts. Analyses were based on the -log10(p) from logistic regression under an additive model of association of SNPs with WBCs. A) A pronounced deviation from the null distribution (red line) is manifested in the model without covariates, λ = 3.99. B) When covariates A_DF_L_PH_B were adjusted in the model λ decreases to 2.76, C) A significant correction is achieved by considering PC1-PC8, λ = 1.31. D) When considering all the covariates together (PC1-PC8_A_H_L_PH_B) the correction level approached the CI 95% of the null distribution (dotted lines), λ = 1.30. (DOCX 429 kb) [file 12864_2018_4523_MOESM4_ESM.docx]

Observed –log_10_(p)

A) λ= 3.99.

B) A_H_L_PH_B λ= 2.76.

C) PC1-PC8 λ= 1.31.

D) PC1-PC8_A_H_L_PH_B λ= 1.30.


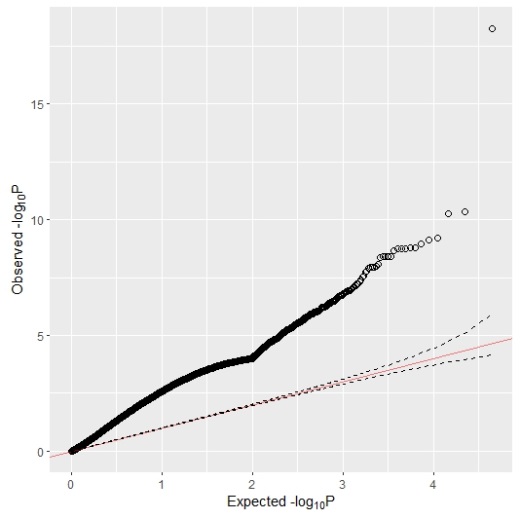

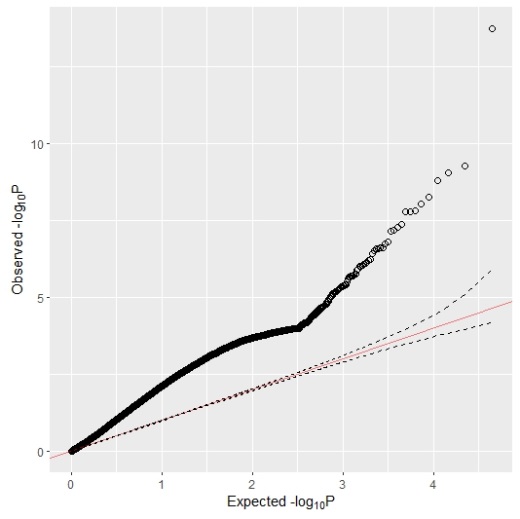

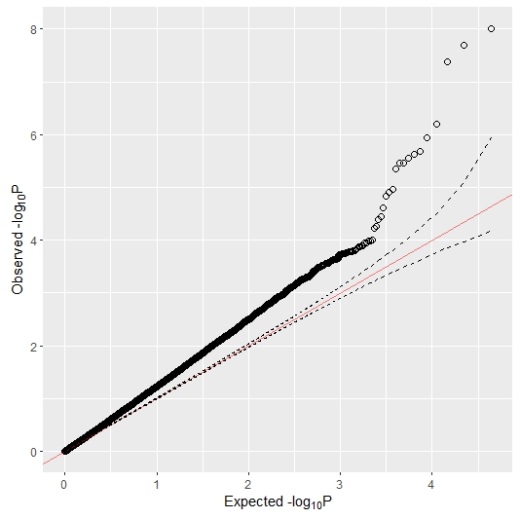

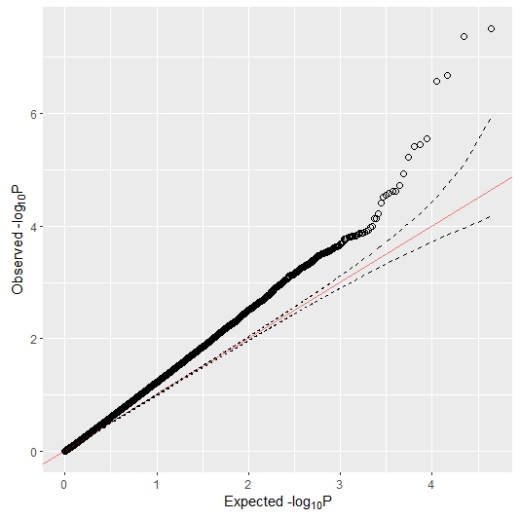


Observed –log_10_(p)

Observed –log_10_(p)

Observed –log_10_(p)

Expected –log_10_(p)

Expected –log_10_(p)

Expected –log_10_(p)

Expected –log_10_(p)
