# Supplementary material for: Genome-wide scan for commons SNPs affecting bovine leukemia virus infection level in dairy cattle
Source: BMC Genomics. 2018 Feb 13;19:142. doi: 10.1186/s12864-018-4523-2 (PMC5812220; doi:10.1186/s12864-018-4523-2)
Supplement: Supplementary file 5 — Table S1. Top 15 SNPs with the lowers p-value from the GWA analysis with PVL excluding SNPs located ~ 20–36 Mb of BTA 23 (DOCX 20 kb). [file 12864_2018_4523_MOESM5_ESM.docx]

Table S1. Top 15 SNPs with the lowers p-value from the GWA analysis with PVL excluding SNPs located ~20-36 Mb of BTA 23.

| **refSNP_ID** |  | **BTA** |  | **Freq** |  | **PVL** | | | | |  | **Position^c^** |
| --- | --- | --- | --- | --- | --- | --- | --- | --- | --- | --- | --- | --- |
|  |  |  |  |  |  | **b^a^** |  | **SD** |  | **p^b^** |  |  |
| rs109905904 |  | 23 |  | 0.12 |  | -0.16 |  | 0.0406 |  | 4.48 x 10-5 |  | 14981935 |
| rs109180964 |  | 12 |  | 0.17 |  | -0.14 |  | 0.0384 |  | 0.00015 |  | 57603061 |
| rs109883939 |  | 23 |  | 0.32 |  | -0.1 |  | 0.0283 |  | 0.00015 |  | 15005574 |
| rs110518606 |  | 23 |  | 0.38 |  | -0.11 |  | 0.0305 |  | 0.00016 |  | 19089496 |
| rs110309928 |  | 23 |  | 0.36 |  | -0.11 |  | 0.0281 |  | 0.00018 |  | 15035028 |
| rs29014446 |  | 23 |  | 0.26 |  | -0.12 |  | 0.0337 |  | 0.00021 |  | 18264611 |
| rs41588613 |  | 23 |  | 0.13 |  | -0.16 |  | 0.0445 |  | 0.00021 |  | 35452881 |
| rs109631491 |  | 13 |  | 0.37 |  | 0.11 |  | 0.0315 |  | 0.00023 |  | 45510831 |
| rs110071620 |  | 23 |  | 0.35 |  | 0.11 |  | 0.0313 |  | 0.00023 |  | 36509046 |
| rs41600189 |  | 23 |  | 0.17 |  | -0.13 |  | 0.0366 |  | 0.00023 |  | 38378023 |
| rs41624517 |  | 11 |  | 0.21 |  | 0.14 |  | 0.0401 |  | 0.00023 |  | 68036682 |
| rs109497003 |  | 13 |  | 0.21 |  | -0.13 |  | 0.0347 |  | 0.00024 |  | 10111504 |
| rs41616835 |  | 11 |  | 0.16 |  | 0.14 |  | 0.0391 |  | 0.00025 |  | 93285949 |
| rs41615922 |  | 28 |  | 0.19 |  | 0.12 |  | 0.0351 |  | 0.00027 |  | 3998395 |

Abbreviations: refSNP_ID: SNP Identification based on dbSNP (http://www.ncbi.nlm.nih.gov/SNP/), Freq: Frequency of the minor allele, PVL: proviral load, SD: standard deviation.

^a^ SNP effect size (β of regression)

^b^ p-value: Under null hypothesis, probability of obtaining the observed effect size.

^c^ SNP location according to bovine genome assembly UMD3.1.
