# Supplementary material for: Genome-wide scan for commons SNPs affecting bovine leukemia virus infection level in dairy cattle
Source: BMC Genomics. 2018 Feb 13;19:142. doi: 10.1186/s12864-018-4523-2 (PMC5812220; doi:10.1186/s12864-018-4523-2)
Supplement: Supplementary file 7 — Table S2. Description of PC categories identified and genes containing them. (DOCX 22 kb). [file 12864_2018_4523_MOESM7_ESM.docx]

Table S2. Description of PC categories identified and genes containing them.

| Name (ID) |  | Definition^a^ |  | Descendants terms^b^ | | | |  | | Genes^c^ | |
| --- | --- | --- | --- | --- | --- | --- | --- | --- | --- | --- | --- |
| Transporter (PC00227) |  | A class of transmembrane proteins that allows substances to cross plasma membranes far faster than would be possible by diffusion alone. Ion channel has its own category and is not included here. |  | - Cation transporter (PC00068) | | - ATP synthase (PC00002) | |  | | *SLC44A4; ATP6V1G2* | |
| Membrane traffic protein (PC00150) |  | A protein that is located on an intracellular vesicle membrane, and is responsible for the docking or fusion of the vesicle to the cytoplasma membrane. |  | - | | - | |  | | *FLOT1* | |
| Hidrolase (PC00121) |  | Enzymes catalyzing hydrolysis of a variety of bonds, such as esters, glycosides, or peptides. Protease and phosphatases are separate categories, so they are not included here. |  | - | | - | |  | | *ATP6V1G2; NEU1* | |
| Enzyme modulator (PC00095) |  | A protein that modulates the activity of a select group of an enzyme such as kinases, phosphatases, protease, and G-proteins. |  | - Kinase modulator (PC00140) | | - | |  | | *BAG6; CSNK2B* | |
| Transcription factor (PC00218) |  | A protein required for the regulation of RNA polymerase by specific regulatory sequences in or near a gene |  | - | | - | |  | | *TFAP2D; PRRC2A* | |
| Ligase (PC00142) |  | A class of enzymes that catalyze the formation of a bond between two substrate molecules, coupled with the hydrolysis of a pyrophosphate bond in ATP or a similar energy donor. |  | - Ubiquitin-protein ligasa (PC00234) | | - | |  | | *TRIM31; TRIM40* | |
| Nucleic acid binding (PC00171) |  | A molecule that binds a nucleic acid. It can be an enzyme or a binding protein. |  | - Nuclease (PC00170) | | - | |  | | *PRRC2A* | |
| Receptor (PC00197) |  | A molecular structure within a cell or on the cell surface characterized by selective binding of a specific substance and a specific physiologic effect that accompanies the binding. |  | - G-protein coupled receptor (PC00021) - Cytokine receptor (PC00084) | | -   - Immunoglobulin receptor superfamily (PC00124) | |  | | *GPR111; BOLA; Ig_like_MHC_I* | |
| Defense/ Immunity protein (PC00090) |  | A specific protein substance that is produced to take part in various defense and immune responses of the body. |  | - Antibacterial response protein (PC00051)      - Immunoglobulin receptor superfamily (PC00124) - Major histocompatibility complex antigen (PC00149) | -    -  - | |  | | *CRISP1; GPR111; BOLA; BOLA-DRB3; Ig_like_MHC_I; BOLA-DQA1* | |  |
| Cytoskeletal protein (PC00085) |  | Major constituent of the cytoskeleton found in the cytoplasm of eukaryotic cells. They form a flexible framework for the cell, provide attachment points for organelles and formed bodies, and make communication between parts of the cell possible. |  | - Actin family cytoskeletal proteins (PC00041) - Microtubule family cytoskeletal protein (PC00157) | | - Actin binding non-motor protein (PC00165) - Tubulin (PC00228) | |  | | *TUBB5; PKHD1* | |
| Signaling molecule (PC00207) |  | A molecule that transduces a signal between cells. |  | - Cytokine (PC00083). | | - Tumor necrosis factor family member (PC00229) | |  | | *LTA; LTB; TNF; CD2AP;TNXB* | |

Abbreviations: ID: Category ID, PC: Panther Protein Class.

^a^ Brief description of the category as Panther (www.pantherdb.org).

^b^ List of names of all descendants terms from a particular PC parent category.

^c^ List of genes assigned to significant SNPs from GWA studies for PVL and WBCs. The following genes did not have a PC associated category: *Olfactory receptors* (ORs), *TRAM2, GCM-1, C23H6ORF47, ABHD16A, LY6G5C, LY6G5B, BAT4, APOM, NFKBIL1, U6, SFTA2, IER3, ABT1* and *NRSN1*.
