# Supplementary material for: Genome-wide scan for commons SNPs affecting bovine leukemia virus infection level in dairy cattle
Source: BMC Genomics. 2018 Feb 13;19:142. doi: 10.1186/s12864-018-4523-2 (PMC5812220; doi:10.1186/s12864-018-4523-2)
Supplement: Supplementary file 6 [file 12864_2018_4523_MOESM2_ESM.docx]

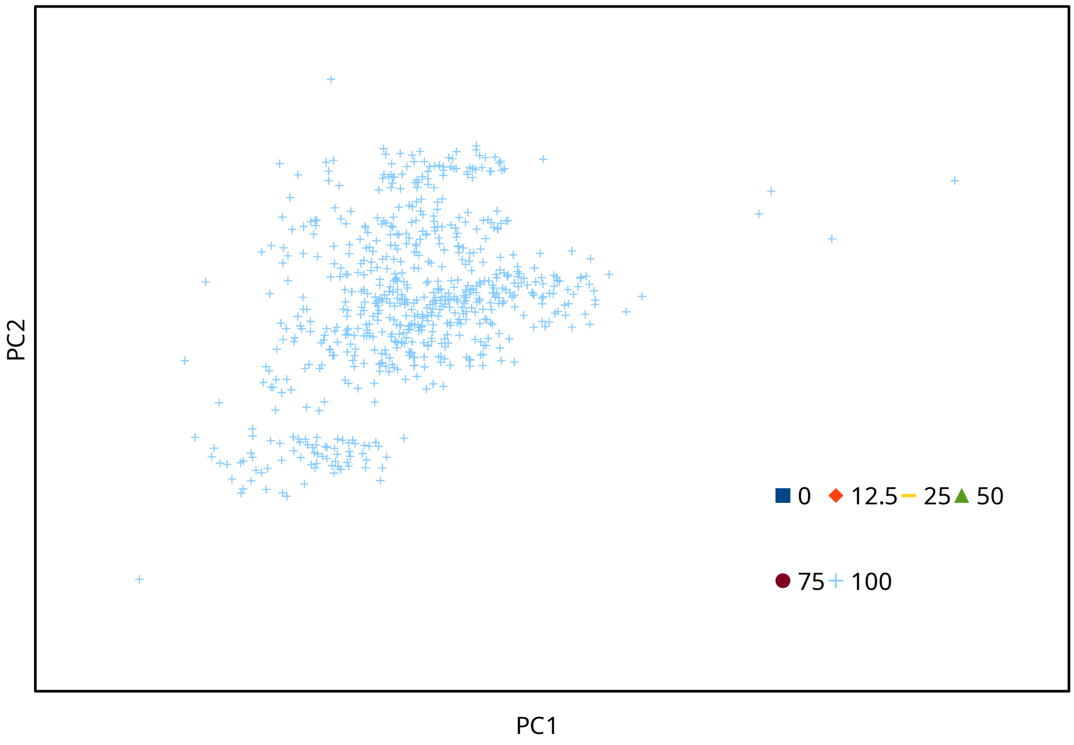

PC2
PC1
0
12.5
25
50
75
100
